# Melissa phospholipids improves sleep quality and mental well-being: concluding results from clinical study in adults with emotional distress

**DOI:** 10.29219/fnr.v70.13729

**Published:** 2026-01-31

**Authors:** Mariangela Rondanelli, Giuseppe Mazzola, Gaetan Claude Barrile, Paola Misiano, Simone Perna

**Affiliations:** 1 Department of Public Health, Experimental and Forensic Medicine, University of Pavia, Pavia, Italy; 2 Endocrinology and Nutrition Unit, Azienda di Servizi alla Persona “Istituto Santa Margherita”, University of Pavia, Pavia, Italy; 3 Department of Pharmacological and Biomolecular Sciences, Università Degli Studi di Milano, Milan, Italy; 4 Division of Human Nutrition, Department of Food, Environmental and Nutritional Sciences (DeFENS), University of Milan, Milan, Italy

**Keywords:** Melissa, lemon balm, sleep quality, emotional well-being, anxiety, emotional distress, Phytosome, phospholipids, rosmarinic acid

## Abstract

**Background:**

*Melissa officinalis* L. (lemon balm) is a botanical support widely used for its calming and sleep-promoting properties.

**Objective:**

To evaluate the impact of daily supplementation with Melissa phospholipids (MOP) at 200 or 400 mg for 3 weeks on sleep quality and psychological well-being in adults with emotional distress and poor sleep quality.

**Design:**

This prospective, open-label, dose-comparison clinical study enrolled 32 adults with poor sleep quality and/or clinically significant emotional distress. Participants received daily supplementation with either 200 or 400 mg/day MOP for 3 weeks. Sleep quality (primary endpoint) was assessed using the Pittsburgh Sleep Quality Index (PSQI) at baseline (T0), and week 3 (T1). Secondary endpoints included depression, anxiety, and stress, positive and negative affect, mental well-being, and quality of life. Safety was also assessed through adherence monitoring, documentation of side effects or adverse events, and by routine blood biochemistry parameters of liver and renal functions.

**Results:**

PSQI scores significantly improved over time (*P* < 0.0001), with greater and earlier benefits at 400 mg/day (−30% vs. −15%; *P* < 0.05). The 400 mg dose also produced significant reductions in depression (−26%), anxiety (−18%), and stress (−22%) scores (all *P* < 0.001), together with increased positive and negative affect together with Warwick-Edinburgh Mental Well-Being Scale (+15%). Quality of life improved across total score (33%) and its four domains, with significant results in physical well-being (*P* < 0.05). No statistically significant changes in safety parameters were detected and no adverse effects were reported.

**Discussion:**

MOP, particularly at 400 mg/day, confirmed to significantly improve sleep and life quality, mood, and overall mental well-being.

**Conclusion:**

These findings support and extend the health benefits of MOP as a well-tolerated, safe natural approach in a dose-dependent frame to managing sleep quality and emotional distress. Future placebo-controlled trials are warranted to confirm these results and further elucidate the underlying mechanisms of action.

## Popular scientific summary

- Melissa phospholipids improved sleep quality in a dose-dependent manner.
- Melissa phospholipids supported psychological relief by improving depression, anxiety, and stress scores.
- Mental well-being and positive affect, and quality-of-life scores improved overall, with significant gains in physical well-being.
- Melissa phospholipids are safe and may offer natural dose-dependent benefits for sleep and emotional distress management. These data extend existing evidence by characterizing both the temporal dynamics of response and the relative impact of different doses.

*Melissa officinalis* L. (lemon balm) is a perennial herbaceous plant widely used in Mediterranean and Asian folk medicine for its calming and mood-stabilizing properties (1).

Its main bioactive constituent, rosmarinic acid, together with other polyphenolic compounds (2) has been shown to modulate central GABAergic neurotransmission, regulate the hypothalamic–pituitary–adrenal (HPA) axis, and exert antioxidant and anti-inflammatory effects in the central nervous system (3–7). Meta-analyses and systematic reviews highlight the anxiolytic and sleep-promoting properties of standardized *M. officinalis* extracts in subjects with anxiety, stress, and insomnia (5, 8). However, most clinical studies are limited by heterogeneity in formulations, short treatment durations, and small sample sizes, and few evaluate both sleep and emotional well-being outcomes in a structured dose-response manner (9).

Recent advances in delivery systems have improved the bioavailability of plant-derived phytochemicals. Food-grade phospholipids-based formulations have demonstrated optimized absorption and greater stability compared with conventional extracts (5, 10–12). Previous pilot studies suggested that 3-week supplementation of *M. officinalis* extract formulated in phospholipids (MOP) could ameliorate sleep quality and emotional distress in adults with poor sleep quality (13–15). Nonetheless, further investigations are warranted to confirm these findings, explore time-dependent patterns of response, and differentiate dose-dependent effects.

Moreover, the bidirectional relationship between poor sleep quality and emotional distress highlights the need to evaluate whether improvements in psychological well-being are mediated primarily by amelioration of sleep disturbances or by direct neuromodulatory actions of *M. officinalis*. Sleep impairment may exacerbate hypothalamic-pituitary-adrenal (HPA) axis hyperactivity, increase systemic inflammation, and impair cognitive and affective regulation, thereby perpetuating emotional distress (5, 7, 16). Conversely, evidence suggests that *M. officinalis* may exert rapid central effects, independently of sleep, through modulation of neurotransmitters and stress-related neuroendocrine pathways (3, 4, 6). Addressing these questions is essential to better delineate the mechanisms underlying clinical effects of MOP supplementation.

The present open-label, prospective clinical study completed and extended previous observations (13) aimed to evaluate the impact of daily supplementation with MOP at 200 mg or 400 mg for 3 weeks, previously proved to be safe (13–15), on sleep quality and psychological well-being in adults with emotional distress and poor sleep quality. Sleep quality, assessed by the Pittsburgh Sleep Quality Index (PSQI), was designated as the primary endpoint, while validated psychometric scales evaluating depression, anxiety, stress (DASS-21), affective states (PANAS), mental well-being (WEMWBS), and quality of life (WHOQoL-BREF) were included as secondary endpoints. The number of subjects was increased (32 vs. 20 compared with the earlier exploratory study), providing new evidence on the clinical utility of *M. officinalis* supplementation in a population at risk for stress-related disorders.

## Materials and methods

### Study design and ethical considerations

This was a prospective, open-label, dose-comparison clinical study conducted in accordance with the ethical standards outlined in the 1964 Declaration of Helsinki and its later amendments, as well as all ICH and ISO international standards (i.e. ICH Harmonized Guideline for GCP E6 (R2); UNI EN ISO 14155:2012), and applicable regulations in Regione Lombardia and national standards of Italy. All participants provided written informed consent prior to enrollment. The clinical study was approved by the Ethics Committee of the University of Pavia (protocol number: 1267/21092023) and registered in a publicly accessible clinical trial registry prior to initiation (ClinicalTrial.gov code NCT06942897).

The study included two parallel intervention arms receiving daily supplementation with 200 or 400 mg/day of Melissa phospholipids/SF (MOP as Melissa Phytosome™, Relissa™) for 3 weeks. Assessments were performed at baseline (T0) and week 3 (T1).

### Study population

Eligible participants were adults aged 18 and 70 years, of either sex, with poor sleep quality defined by a PSQI score greater than 5 and/or clinically significant emotional distress. Emotional distress was confirmed by scores above 14, 10, and 19 on the depression, anxiety, and stress subscales of the DASS-21 questionnaire, respectively. Exclusion criteria included the use of benzodiazepines, antidepressants, or other psychotropic medications within the study period, the presence of primary sleep disorders such as obstructive sleep apnea or restless legs syndrome, significant neurological, psychiatric, or cardiovascular comorbidities, pregnancy or lactation, and a known hypersensitivity to *M. officinalis* or phospholipid-based supplements.

**Fig. 1:**
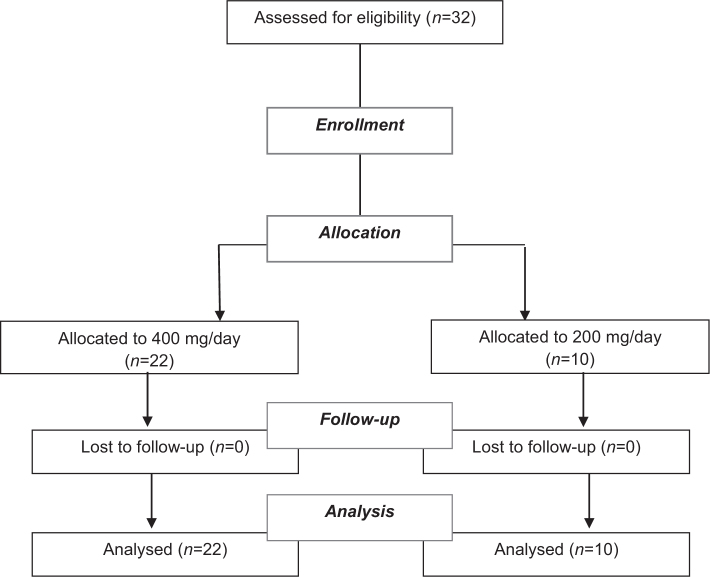
Flow diagram of the study.

A total of 32 participants meeting the inclusion criteria were enrolled. Ten participants were assigned to receive 200 mg/day and 22 participants to the 400 mg/day dose. Allocation was based on chronological order of enrollment, as the study was designed as open parallel arms, as shown in the flow chart in Figure 1.

### Intervention

The participants received supplementation for 3 weeks with one or two 200 mg tablets once daily after dinner containing MOP. Each tablet contained 200 mg of *M. officinalis* extract. Participants were instructed to take one or two tablets in the evening, approximately 30 to 60 min before bedtime, with a glass of water. Treatment adherence was monitored at each follow-up visit through direct questioning and tablet count.

### Endpoints

The primary endpoint of the study was the change in sleep quality over time, assessed by the PSQI total score (17) measured at baseline and week 3. Secondary endpoints included changes in emotional distress, measured by the depression, anxiety, and stress subscales of the DASS-21 questionnaire (18), changes in affective states using the Positive and Negative Affect Schedule (PANAS POS and PANAS NEG) (19), changes in mental well-being using the Warwick-Edinburgh Mental Well-Being Scale (WEMWBS), and changes in quality of life using the WHO Quality of Life-BREF (WHOQoL-BREF) total score and its four domains: physical health, psychological health, social relationships, and environment (20). All validated questionnaires were administered by trained research staff following standardized procedures.

### Assessment of safety

The biochemical parameters were assessed at T0 and T1. Venous blood samples were drawn after an overnight fast. Blood samples were obtained through an indwelling catheter inserted in an antecubital vein, and were immediately centrifuged and stored at −80°C until assayed.

For the assessment of safety, routine blood biochemistry parameters of liver and renal function were evaluated: creatinine, alanine aminotransferase, aspartate aminotransferase, and gamma glutamyl transferase were measured with enzymatic–colorimetric methods. Moreover, fasting blood glucose (FBG), total cholesterol (TC), low-density lipoprotein cholesterol (LDL-C), high-density lipoprotein cholesterol (HDL-C), and triglyceride (TG) levels were measured by an automatic biochemical analyzer (Hitachi 747, Tokyo, Japan).

### Statistical analysis

Data for each variable were statistically evaluated by 2-way (treatment, time) ANOVA (Analysis Of Variance) with repeated treatment on the factor ‘time’, followed by Sidak’s test for post hoc analysis. Within the blood biochemistry subset, changes over time (primarily T0 vs. T1) were analyzed using the paired *t*-test. Variables are reported as mean ± mean standard error (SEM). Statistical significance was defined as a two-tailed *P*-value < 0.05. Missing data, which represented less than 5% of the total dataset, were imputed using the last observation carried forward (LOCF) method.

## Results

A total of 32 participants completed the 3-week supplementation period, 10 assigned to the 200 mg/day group and 22 assigned to the 400 mg/day group. No participants discontinued supplementation or were lost to follow-up during the study.

Baseline demographic and clinical characteristics are summarized in Table 1. The two groups were well balanced with respect to age, sex distribution, and Body Mass Index (BMI). Likewise, there were no statistically significant differences at baseline between the groups in PSQI total scores, DASS-21 subscale scores, PANAS Positive and Negative, WEMWBS scores, or WHOQoL-BREF total and domain scores.

**Table 1:** Baseline demographic and clinical characteristics of the study population at T0

Characteristic	200 mg/day (*n* = 10)	400 mg/day (*n* = 22)
Age (years)	51.9 ± 2.27	51.4 ± 1.42
Sex (M/F)	50% (5)/50% (5)	50% (11)/50% (11)
BMI (kg/m²)	25.49 ± 0.41	25.67 ± 0.50
PSQI total score	8.80 ± 0.71	8.36 ± 0.42
DASS-21 Depression	21.30 ± 1.13	21.86 ± 0.57
DASS-21 Anxiety	20.60 ± 0.91	20.27 ± 0.40
DASS-21 Stress	21.10 ± 0.77	21.32 ± 0.36
PANAS Positive	14.10 ± 0.53	14.05 ± 0.32
PANAS Negative	15.30 ± 0.54	15.45 ± 0.23
WEMWBS total score	38.80 ± 1.17	38.32 ± 0.62
WHOQoL-BREF total score	5.30 ± 0.54	5.14 ± 0.30

Values are expressed as mean ± SEM or number (%).

Overall, the baseline data confirmed that the two groups were similar across all primary and secondary outcome measures, providing a consistent foundation for evaluating supplementation-related changes over time.

### Effects on sleep quality (PSQI)

**Fig. 2:**
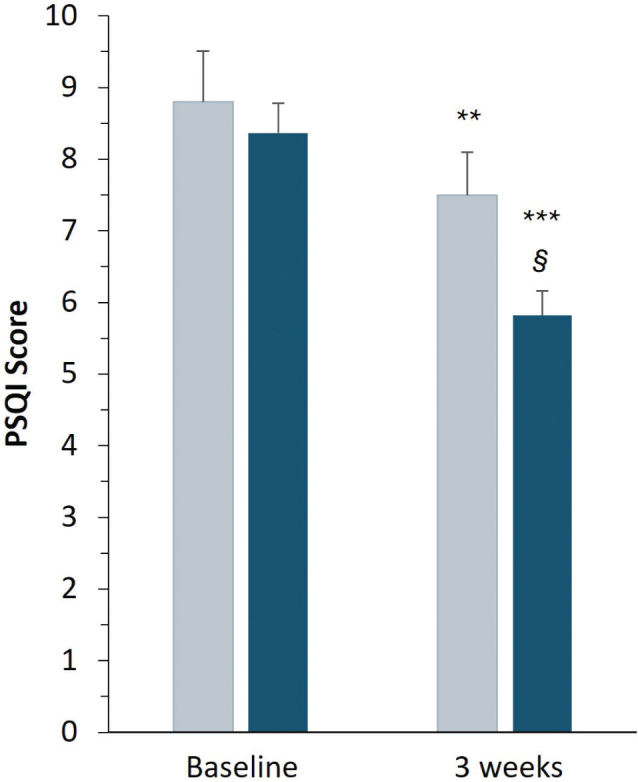
Effects of sleep quality as Pittsburgh Sleep Quality Index (PSQI) score of Melissa phospholipids at 200 mg (gray) or 400 mg (blue) daily at baseline (T0) or after 3-week supplementation (T1). Bars represent mean PSQI values with the mean standard error. A *P*-value of < 0.05 was considered statistically significant. **P < 0.005 and ***P < 0.0001, significant intra-group differences T1 versus baseline; §*P* < 0.05, significant group differences at T1, by Sidak’s post hoc tests.

Figure 2 and Table 2 summarize the changes in PSQI total scores from baseline (T0) to week 3 (T1) in the two treatment groups. Two-way repeated-measures ANOVA revealed a significant main effect of time (*P* < 0.0001) and a significant time × treatment interaction (*P* < 0.01), indicating that the magnitude and timing of improvement differed between doses.

**Table 2:** Changes in Pittsburgh Sleep Quality Index (PSQI) total scores from baseline (T0) to week 3 (T1)

Time point	200 mg/day (*n* = 10)	% change vs. baseline	400 mg/day (*n* = 22)	% change vs. baseline	*P*-value (between groups)
Baseline (T0)	8.80 ± 0.71	–	8.36 ± 0.42	–	*P* > 0.05
Week 3 (T1)	7.50 ± 0.60 **	−14.77%	5.82 ± 0.35**	−30.38%	***P* < 0.05**
Post hoc comparisons (Sidak’s test) *P*-values T0 vs. T1	***P* < 0.005**		***P* < 0.0001**		

Values are expressed as mean ± SEM. Two-way repeated-measures ANOVA revealed a significant main effect of time (*P* < 0.0001) and a significant time × treatment interaction (*P* < 0.01). Sidak’s post hoc tests were used for pairwise comparisons.

In the 200 mg/day group, PSQI scores showed a gradual decrease, from 8.80 at baseline to 7.50 after 3 weeks (−14.8% from baseline; *P* < 0.005, Sidak’s post hoc test). Participants receiving 400 mg/day exhibited a faster and more pronounced improvement in sleep quality. Mean PSQI scores decreased significantly from 8.36 at baseline to 5.82 at week 3, corresponding to −30.4% from baseline (*P* < 0.0001). Between-group comparison at week 3 confirmed that the higher dose produced significantly greater improvement compared with the 200 mg/day regimen (*P* < 0.05). Furthermore, the proportion of participants achieving a clinically meaningful reduction in PSQI score (≥ 3 points from baseline) was notably higher in the 400 mg/day group than the 200 mg/day group (54.5% vs. 30%, respectively) at the end of the study period. Overall, *M. officinalis* phospholipids supplementation was associated with a clinically and statistically significant improvement in sleep quality, with the 400 mg/day dose eliciting an earlier and more substantial effect than the 200 mg/day dose.

### Effects on mental well-being

Table 3 summarizes the changes in DASS-21 scores for depression, anxiety, and stress from baseline (T0) to week 3 (T1), including two-way repeated-measures ANOVA followed by post hoc comparisons between time points and treatment groups.

**Table 3:** Changes in DASS-21 scores (Depression, Anxiety, Stress) from baseline (T0) to week 3 (T1)

Subscale & time point	200 mg/day (*n* = 10)	% chang vs. baseline	400 mg/day (*n* = 22)	% change vs. baseline	*P*-value (between groups)
Depression / Total scores					
Baseline (T0)	21.30 ± 1.13	–	21.86 ± 0.57	–	*P* > 0.05
Week 3 (T1)	19.90 ± 0.94	−6.10%	16.09 ± 0.83	−26.39%	***P* < 0.01**
Post hoc comparisons (Sidak’s test) *P*-values T0 vs. T1	*P* > 0.05		***P* < 0.0001**		
Anxiety scores					
Baseline (T0)	20.60 ± 0.91	–	20.27 ± 0.40	–	*P* > 0.05
Week 3 (T1)	19.20 ± 0.79	−6.80%	16.68 ± 0.54	−17.71%	***P* < 0.05**
Post hoc comparisons (Sidak’s test) *P*-values T0 vs. T1	*P* > 0.05		***P* < 0.0001**		
Stress scores					
Baseline (T0)	21.10 ± 0.77	–	21.32 ± 0.36	–	*P* > 0.05
Week 3 (T1)	19.00 ± 1.13	−9.95%	16.68 ± 0.43	−21.76%	***P* < 0.05**
Post hoc comparisons (Sidak’s test) *P*-values T0 vs. T1	***P* < 0.05**		***P* < 0.0001**		

Changes in DASS-21 scores (Depression, Anxiety, Stress) from baseline (T0) to 1 week 3 (T1) in the 200 mg/day and 400 mg/day groups. Values are expressed as mean ± SEM. Two-way repeated-measures ANOVA was used to assess the effects of time, treatment, and their interaction. When significant, Sidak’s post hoc tests were performed for pairwise comparisons.

For depression, participants receiving 400 mg/day of *M. officinalis* phospholipids showed a robust and highly significant reduction in scores, decreasing −26.4% after week 3 (*P* < 0.0001 vs. baseline). In contrast, the 200 mg/day group exhibited only a modest, non-significant decrease of −6.1% (*P* > 0.05). The between-group comparison at week 3 confirmed a significant difference in favor of the higher dose (*P* < 0.01), indicating a clear dose–response relationship.

Regarding anxiety, mean scores decreased significantly in the 400 mg/day group (from 20.27 to 16.68; −17.7%; *P* < 0.0001), while the reduction in the 200 mg/day group was smaller and not statistically significant (−6.8%; *P* > 0.05). Between-group analysis at week 3 confirmed a significant advantage for the 400 mg/day regimen (*P* < 0.05).

For stress, a significant improvement was observed at both dosages. The 400 mg/day group exhibited a marked reduction after 3 weeks of −21.8% (*P* < 0.0001), while the 200 mg/day group showed a smaller but statistically significant decrease of −9.9% (*P* < 0.05). Between-group comparison again favored the higher dose (*P* < 0.05).

These findings demonstrate that *M. officinalis* phospholipids supplementation exerted clinically relevant improvements across mood-related domains, with a consistent dose–response pattern. The 400 mg/day dose produced broader and more pronounced benefits after 3 weeks of supplementation, whereas the 200 mg/day regimen yielded measurable but smaller effects, reaching significance only for the reduction in perceived stress.

Table 4 reports the changes in PANAS scores for positive and negative affect from baseline (T0) to week 3 (T1), along with post hoc comparisons between time points and treatment groups.

**Table 4:** Changes in PANAS scores (Positive and Negative Affect) from baseline (T0) to week 3 (T1)

Subscale & Time point	200 mg/day (*n* = 10)	% change vs. baseline	400 mg/day (*n* = 22)	% change vs. baseline	*P*-value (between groups)
PANAS POS					
Baseline (T0)	14.10 ± 0.53	-	14.05 ± 0.32	-	*P* > 0.05
Week 3 (T1)	15.90 ± 0.59	+12.77%	16.55 ± 0.40	+17.79%	NA
Post hoc comparisons (Sidak’s test) *P*-values T0 vs. T1	NA		NA		
PANAS NEG					
Baseline (T0)	15.30 ± 0.54	−	15.45 ± 0.23	−	*P* > 0.05
Week 3 (T1)	14.00 ± 0.60	−8.50%	12.68 ± 0.31	−17.80%	***P* < 0.05**
Post hoc comparisons (Sidak’s test) *P*-values T0 vs. T1	***P* < 0.01**		***P* < 0.0001**		

Values are expressed as mean ± SEM. Two-way repeated-measures ANOVA was used to assess the effects of time, treatment, and their interaction. Sidak’s post hoc tests were used for pairwise comparisons between time points.

From baseline to week 3, positive affect increased in both groups, with a more positive trend respecting time after 400 mg/day supplementation (+17.8% vs. baseline).

For negative affect, scores decreased over the same period, indicating reduced irritability and tension. The 200 mg/day group showed a moderate but significant reduction from 15.30 to 14.00 (−8.5%; *P* < 0.01), whereas the 400 mg/day group exhibited a more pronounced decrease from 15.45 to 12.68 (−17.8%; *P* < 0.0001). The between-group comparison at week 3 confirmed a significant advantage for the higher dose (*P* < 0.05).

Overall, supplementation with *M. officinalis* phospholipids produced a favorable modulation of affective states, enhancing positive mood and reducing negative emotionality. Both dosages were effective, but the 400 mg/day regimen was associated with broader and more substantial improvements in affective balance.

Table 5 summarizes the changes in Mental Well-being assessed by WEMWBS scores from baseline (T0) to week 3 (T1) in both groups. Two-way repeated-measures ANOVA revealed a significant main effect of time (*P* < 0.0001) and a significant time × treatment interaction (*P* < 0.01), indicating that the magnitude of improvement differed between the two doses.

**Table 5:** Changes in Warwick-Edinburgh Mental Well-being Scale (WEMWBS) and WHO Quality of Life BREF (WHOQoL-BREF) scores from baseline (T0) to week 3 (T1)

Score and Time point	200 mg/day (*n* = 10)	% change vs. baseline	400 mg/day (*n* = 22)	% change vs. baseline	*P*-value (between groups)
WEMWBS					
Baseline (T0)	38.80 ± 1.17	–	38.32 ± 0.62	–	*P* > 0.05
Week 3 (T1)	40.10 ± 1.27	+3.35%	44.18 ± 0.85	+15.29%	***P* < 0.01**
Post hoc comparisons (Sidak’s test) *P*-values T0 vs. T1	*P* > 0.05		***P* < 0.0001**		
WHOQoL-BREF Total score					
T0	5.30 ± 0.45	-	5.14 ± 0.30	-	*P* > 0.05
T1	5.70 ± 0.54	+7.54%	6.82 ± 0.28	+32.68%	*P* > 0.05
Post hoc comparisons (Sidak’s test) *P*-values T0 vs. T1	*P* > 0.05		***P* < 0.0001**		

Values are expressed as mean ± SEM. Statistical analysis was performed using two-way repeated-measures ANOVA and Sidak’s post hoc tests.

In the 200 mg/day group, mean WEMWBS scores increased modestly to a +3.35% from baseline to week 3. However, this variation did not reach statistical significance (*P* > 0.05, Sidak’s post hoc test). In contrast, participants receiving 400 mg/day of *M. officinalis* phospholipids showed a marked and significant enhancement in perceived mental well-being, with mean scores rising from 38.32 to 44.18 after 3 weeks (+15.29%; *P* < 0.0001). The between-group comparison at week 3 confirmed a significant advantage for the 400-mg dose (*P* < 0.01), indicating a clear dose–response relationship in the improvement of overall psychological well-being.

Overall, MOP supplementation produced a significant and clinically relevant increase in mental well-being at the 400 mg/day dosage, while the lower dose induced only minor, non-significant changes over the same period.

The WHOQoL-BREF total score is summarized in Table 5, as the changes observed in the WHOQoL-BREF questionnaire from baseline (T0) to week 3 (T1). Two-way repeated-measures ANOVA was applied to assess time, treatment, and interaction effects, followed by Sidak’s post hoc comparisons.

In the 200 mg/day group, the total score remained almost stable from baseline (5.30) to week 3 (5.70) resulting in a modest improvement of +7.54% (*P* > 0.05). In the 400 mg/day group, the total score significantly improved from baseline to week 3 of +32.68% (*P* < 0.0001). At week 3, the total score was numerically higher in the 400 mg/day group compared with the 200 mg/day group, but the difference did not reach statistical significance (*P* = 0.083).

Across the four domains of WHOQoL BREF, the most pronounced improvements were observed in the 400 mg/day group (data not shown). In the Physical Health Domain, mean scores increased in both treatment groups, with a greater magnitude in participants receiving the higher dose. In the 200 mg/day group, scores rose from 19.90 ± 1.13 at baseline to 21.10 ± 0.96 at week 3 (+6.0%; *P* > 0.05). In contrast, the 400 mg/day group exhibited a marked improvement from 20.18 ± 0.55 to 23.45 ± 0.33 (+16.2%; *P* < 0.0001). The between-group comparison at week 3 confirmed a significant advantage for the 400 mg/day regimen (*P* < 0.05), suggesting a dose-dependent enhancement in physical well-being.

The Mental Health Domain also improved over time during the 3-week supplementation. Participants receiving 200 mg/day showed an increase of +14.7%, while those in the 400 mg/day group experienced a greater improvement of +23.6% from baseline. Although formal between-group comparisons were not available, the overall trend indicates a consistent and clinically meaningful enhancement in mental well-being, particularly at the 400-mg dose.

Improvements were also observed in the Interpersonal Relationships Domain. In the 200 mg/day group, scores increased to +20.7% after 3 weeks, while in the 400 mg/day group they rose to a +25.9%. Although between-group comparisons were not statistically assessed, the higher dose was associated with a better overall improvement in perceived relational quality.

Finally, the Environment Domain showed parallel benefits. Scores increased to a +9.4% (*P* > 0.05) in the 200 mg/day group, and to a +12.6% (*P* < 0.005) in the 400 mg/day group. While between-group differences were not calculated, the higher dosage again produced a more consistent and marked improvement.

### Safety and tolerability

Table 6 reports the parameters of safety evaluated. No statistically significant changes in these safety parameters were detected. Also metabolic parameters (lipidic and glycemic parameters) did not change over time (data not shown).

**Table 6:** Blood biochemistry parameters from baseline (T0) to week 3 (T1)

200 mg (*n* = 10)	AST		ALT		GGT		Creatinine	
	T0	T1	T0	T1	T0	T1	T0	T1
Mean	18.3	18.6	21.6	20.5	21.2	20.1	0.84	0.85
SEM	1.27	1.85	1.51	1.67	5.12	4.54	0.04	0.04
*P*-value T1 vs. T0		0.840		0.409		0.287		0.794
**400 mg (*n* = 22)**	**AST**		**ALT**		**GGT**		**Creatinine**	
	**T0**	**T1**	**T0**	**T1**	**T0**	**T1**	**T0**	**T1**
Mean	17.32	16.91	24.05	26.27	21.14	22.59	0.77	0.77
SEM	1.00	1.02	2.83	2.99	2.95	3.70	0.02	0.03
*P*-value T1 vs. T0		0.645		0.399		0.570		0.939

Values are expressed as mean ± SEM. Statistical analysis was performed using the paired *t*-test.

Moreover, MOP supplementation was generally well tolerated throughout the study, no adverse events were reported, and no withdrawals occurred during the study. Overall adherence to the study intervention was high. Compliance with supplementation, assessed by tablet counts and participant reporting, exceeded 95% in both doses’ groups.

## Discussion

This study demonstrates that 3 weeks of daily supplementation with a standardized phospholipid-based *M. officinalis* extract was associated with meaningful improvements in sleep quality and multiple domains of psychological well-being in adults experiencing emotional distress and poor sleep quality. The effects were consistently more pronounced in participants receiving the 400 mg/day dose compared with those receiving 200 mg/day, suggesting a dose-response relationship.

Sleep quality, assessed as the primary endpoint through the PSQI total score, improved significantly in both supplemented groups, with a greater reduction observed at the higher dose. Participants receiving 400 mg/day showed a more than twofold greater reduction in PSQI scores after 3 weeks, compared with the observed high decrease in the 200 mg/day group. These findings are consistent with previous studies reporting the sleep-promoting properties of *M. officinalis* extracts (3–5) and extend current evidence by demonstrating a clear dose-dependent effect using a phospholipid-based formulation. Beyond sleep, significant benefits were observed in psychological domains. Depression, anxiety, and stress scores (DASS-21) decreased substantially in the 400 mg/day group, accompanied by corresponding improvements in positive affect and a significant reduction in negative affect (PANAS). Similarly, mental well-being (WEMWBS) improved significantly at the higher dose, while the 200 mg/day regimen produced only modest, not-significant changes. Quality of life, assessed by the WHOQoL-BREF, also improved in total scores and across multiple domains, with particularly notable effects in physical health domain. These results collectively indicate that MOP exert broad psychotropic benefits that extend beyond sleep regulation. The question of whether these improvements are primarily mediated by enhanced sleep quality or by direct neuropsychological effects of *M. officinalis* remains open. The parallel improvement in both sleep and affective measures supports a synergistic model, where enhanced sleep contributes to emotional stabilization, while the extract’s intrinsic neuroactive properties further alleviate mental distress. This interpretation is consistent with the well-established bidirectional relationship between sleep disturbance and emotional dysregulation, in which sleep impairment amplifies stress reactivity and depressive symptomatology (21, 22). From a mechanistic standpoint, preclinical evidence supports a multifaceted mode of action for *M. officinalis*, particularly through its polyphenolic components such as rosmarinic acid, which modulate GABAergic transmission, attenuate HPA axis hyperactivity, and exert anti-inflammatory and antioxidant effects (3–5). Recent in vitro studies have confirmed the non-cytotoxic profile in neuronal cells, the antioxidant and neuroprotective properties of the same phospholipid-based formulation used in this study (6), while clinical data from controlled trials have demonstrated benefits in insomnia and stress-related symptoms (14, 15).

Moreover, no safety or tolerability concerns occurred during the study, confirming the good compliance of the supplementation and the safe profile of MOP at doses and schedule used.

Overall, the present findings demonstrate that daily supplementation with a phospholipid-based *M. officinalis* extract at 400 mg induces clinically meaningful improvements in sleep quality, mood regulation, and overall psychological well-being within a short intervention period of 3 weeks. The 200 mg/day dosage also showed beneficial trends, although of smaller magnitude and without statistical significance for several endpoints. Despite the limitations related to the open-label design, small sample size, and short duration, these results support the clinical relevance of *M. officinalis* phospholipid supplementation, particularly at 400 mg/day, as a natural short-term, well-tolerated approach for individuals experiencing emotional distress and sleep disturbances.

Some limitations of this study warrant consideration. The open-label design and the absence of a placebo-controlled group limit the ability to exclude expectancy and observer effects. The small sample size, particularly in the group supplemented with 200 mg daily, reduces statistical power and constrains the generalizability of the results. The supplementation period was relatively short, lasting only 3 weeks, which precludes conclusions regarding long-term persistence or sustainability of the observed benefits.

These limitations highlight key directions for future research. There is, firstly, a clear need for randomized, double-blind, placebo-controlled clinical trials with larger sample sizes to confirm the effects of *M. officinalis* supplementation on sleep quality and psychological well-being. Some previous randomized, double blind, placebo-controlled trials have already reported the potential benefits of MOP (14, 15). Nonetheless, new trials should be adequately powered to detect clinically meaningful differences between doses and to minimize expectancy effects inherent to open-label designs, which were not addressed here. In addition, future studies should incorporate longer follow-up periods to evaluate the persistence and trajectory of improvements beyond the initial 3 weeks of supplementation. This would clarify whether the observed benefits plateau, continue to progress, or diminish with extended supplementation period. Longer interventions may also help to determine whether repeated or cyclical supplementation strategies, likely integrating objective sleep assessments, such as actigraphy and polysomnography, provide enhanced benefits for individuals with recurrent sleep disturbances and emotional distress, as preliminarily reported for MOP (15).

Finally, future research should systematically include validated neuroendocrine and inflammatory biomarkers, such as salivary cortisol profiles, cytokine panels, and neurotrophic factors, in both dosage groups and across the entire study population. This comprehensive approach would enable a deeper exploration of the biological mechanisms underpinning the observed clinical benefits and help to clarify the dose–response relationship between MOP supplementation and stress regulation.

## Conclusions

This study demonstrates that 3 weeks of daily supplementation with a standardized phospholipid-based *M. officinalis* extract leads to clinically meaningful improvements in sleep quality and multiple domains of mental well-being in adults with emotional distress and poor sleep quality. The effects were dose-dependent, with the 400 mg/day regimen producing earlier and more pronounced benefits compared with the 200 mg/day dose. These findings support and extend prior evidence of the sleep-promoting and anxiolytic properties of MOP reported particularly at 400 mg/day, and further extend the current literature by characterizing both the temporal dynamics of response and the relative impact of different doses.

## Data Availability

The data presented in this study are available on request from the corresponding author.
